# Nanomechanical and Adhesive Behavior of Electrophoretically Deposited Hydroxyapatite- and Chitosan-Based Coatings on Ti13Zr13Nb Alloy

**DOI:** 10.3390/ma18235323

**Published:** 2025-11-26

**Authors:** Michał Bartmański

**Affiliations:** Department of Biomaterials Technology, Faculty of Mechanical Engineering and Ship Technology, Gdańsk University of Technology, 80-233 Gdańsk, Poland; michal.bartmanski@pg.edu.pl; Tel.: +48-500-034-220

**Keywords:** nanoindentation, nanoscratch-test, hydroxyapatite, chitosan, surface modification, titanium alloy

## Abstract

This work reports on the effects of surface pre-treatment and EPD process parameters on the nanomechanical and adhesive performance of chitosan-based composite coatings fabricated on a Ti13Zr13Nb alloy. Three different coating systems were prepared: chitosan–Cu (series A), chitosan–HAp (series B), and HAp–Cu (series C). Coatings were deposited from suspensions at different voltages (10–30 V) and for various times (1–2 min) onto polished, anodized, and laser surface-treated titanium alloy substrates. Microstructural, nanomechanical, and adhesion properties were characterized by means of SEM, nanoindentation, and nanoscratch testing, respectively. Chitosan–Cu coatings exhibited the highest hardness (up to 8.2 GPa) and stiffness due to the homogeneous dispersion of Cu nanoparticles and strong interfacial bonding to the underlying anodized TiO_2_ layer. Chitosan–HAp coatings were softer (0.05–0.13 GPa) and highly plastic, particularly after laser surface treatment due to their specific porous, polymer-dominated structure. HAp–Cu coatings exhibited an intermediate mechanical behavior with a hardness between 0.1 GPa and 2.9 GPa and enhanced elastic recovery (Wp/We ≈ 3.5–4.7), particularly for anodized substrates. The nanoscratch test results showed that the HAp–Cu coatings exhibited the highest adhesion Lc (≈150–173 mN), confirming a synergistic effect of hybrid composition and heat treatment on interfacial toughness. The present data demonstrate that the optimization of anodizing and EPD processing parameters allows for the manipulation of the mechanical integrity and adhesion of bioactive chitosan-based coatings for titanium biomedical applications.

## 1. Introduction

Titanium and its alloys are still considered the optimal choice for implants that bear mechanical loads because of their excellent corrosion resistance, good biocompatibility, and appropriate mechanical properties [1,2]. Among these, the β-type Ti13Zr13Nb alloy has been receiving special attention due to its low elastic modulus and because toxic alloying elements such as Al and V, which are present in the traditionally used Ti6Al4V alloy, are absent [3]. On the other hand, the bioinert nature of its naturally formed oxide layer limits osseointegration, motivating the development of surface treatments aimed at improving biological activity and long-term implant stability.

Chitosan, as a natural polysaccharide, has recently been widely investigated as a biofunctional coating material due to its biocompatibility, biodegradability, and antimicrobial potential [4]. However, its rather low hardness and limited adhesion to metallic substrates prevent chitosan from being directly used for load-bearing applications. In order to enhance these characteristics, chitosan can be reinforced either by inorganic or metallic nanoparticles, forming hybrid coatings with a higher stiffness and durability [5]. Hydroxyapatite (HAp) is a calcium phosphate compound that shares chemical similarities with bone mineral and increases osteoconductivity, whereas the addition of metallic nanoparticles, such as copper (Cu), may exhibit antibacterial properties and induce additional particle–matrix reinforcement effects [6,7].

Electrophoretic deposition has proven to be a versatile and low-cost technique for developing such hybrid coatings on titanium alloys [8,9]. This technique offers the advantages of accurately controlling thickness, homogenous particle distributions, and processing at a low temperature that maintains the biological functionality of organic and bioactive components [10]. In general, coating quality and performance depend strongly on several factors such as suspension composition, voltage, deposition time applied, and surface pre-treatment [11]. The anodization of titanium substrates forms a porous TiO_2_ nanotubular layer, enhancing mechanical interlocking and adhesion, while laser modification can alter the morphology of oxides and have an influence on the nucleation during EPD [12,13].

Nanoindentation provides a key contribution to the development of thin coatings intended for biomedical applications, with the mechanical integrity at the nanoscale level of the coating–substrate system determining its ability to bear the functional stresses imposed by micromotion, wear, and fatigue [14,15,16]. It provides quantitative values of hardness (H) and Young’s modulus (E) from load–displacement curves, which give evidence on the resistance to plastic deformation and the elastic recovery of the material. Moreover, nanoindentation mapping enables the visualization of property gradients and local mechanical heterogeneity across the coating thickness, which is crucial for layered systems such as biofunctional coatings on titanium alloys. The analysis of nanomechanical parameters such as H/E, H^3^/E^2^, and Wp/We ratios gives valuable insight into coating stiffness, toughness, and elastic–plastic balance—parameters directly related to adhesion and in-service durability [17,18]. In this context, nanoindentation together with nanoscratch testing represents a powerful methodology to correlate microstructural features with their mechanical performances and to identify optimal coating configurations for biomedical implant applications [19].

Consequently, the current work deals with the influence of surface pre-treatment (grinding, anodization, laser modification) and electrophoretic deposition parameters (voltage and time) on the structural, nanomechanical, and adhesive properties of chitosan-based coatings containing copper nanoparticles, hydroxyapatite, and their hybrid combinations deposited on a Ti13Zr13Nb alloy.

## 2. Materials and Methods

### 2.1. Substrate Material Preparation

The Ti13Zr13Nb alloy (SeaBird Metal Materials Co., Baoji, China) employed as the substrate possessed the chemical composition presented in Table 1. Disk-shaped specimens 4 mm in thickness were sectioned from rods with a diameter of 20 mm. The surfaces were mechanically ground using a series of abrasive papers with grid no. 800 (for laser treatment) and no. 2000 as the final step, resulting in an average surface roughness of Sa = 0.13 µm. Subsequently the specimens were ultrasonically cleaned (Sonic-3. POLSONIC, Warsaw, Poland) for 15 min in pure isopropanol (Polskie Odczynniki Chemiczne POCH, Gliwice, Poland) followed by deionized water to remove any residual contaminants.

### 2.2. Electrochemical Oxidation of Ti13Zr13Nb Alloy

Electrochemical oxidation was performed in an electrolyte composed of 10 mL of 85% orthophosphoric acid (1 M H_3_PO_4_; Sigma-Aldrich, St. Louis, MO, USA), 1.2 mL of 40% hydrofluoric acid (HF; Polskie Odczynniki Chemiczne POCH, Gliwice, Poland), and 150 mL of deionized water. The process was conducted using a standard electrochemical setup consisting of an electrochemical cell connected to a DC power supply (MCP/SPN110-01C. Shanghai MCP Corp., Shanghai, China) with a platinum electrode serving as the cathode and the Ti13Zr13Nb alloy specimen as the anode positioned at a distance of 10 mm. Oxidation was carried out at room temperature under a constant voltage of 20 V for 20 min. Following the treatment the specimens were thoroughly rinsed with distilled water and air-dried at ambient temperature for 24 h.

### 2.3. Laser Treatment

Samples polished up to a grit size of 800 were subjected to laser surface treatment using a pulsed Nd:YAG laser (TruLaser Station 5004. TRUMPF, Ditzingen, Germany) under a protective argon atmosphere. The entire surface of each specimen was scanned by the laser beam. The laser parameters were as follows: beam power of 750 or 1500 W, pulse duration of 3.25 ms, scanning speed of 6 mm/s, and pulse frequency of 25 Hz.

### 2.4. Coatings Deposition

The coatings were deposited in Procedures A, B, and C. All details of surface modifications applied in various procedures are presented on Table 2 and described in Section 2.4.1, Section 2.4.2 and Section 2.4.3.

#### 2.4.1. Procedure A—Chitosan–nanoCu Coatings

The electrolytes were prepared by dispersing 1 g of high-molecular-weight chitosan (degree of deacetylation > 75%; Sigma-Aldrich, St. Louis, MO, USA) in 1 L of 1% (*v*/*v*) acetic acid (Polskie Odczynniki Chemiczne POCH, Gliwice, Poland). Two types of electrolytes were prepared—one without and one with 1 mL of Tween 20 (Polysorbate 20; Sigma-Aldrich, St. Louis, MO, USA) diluted in 1 L of 1% acetic acid. The solutions were homogenized using a magnetic stirrer at 250 rpm for 24 h at room temperature. One hour prior to electrophoretic deposition (EPD), 0.05 g/L of copper nanopowder (Hongwu International Group Ltd., Guangzhou, China) with an average particle size of approximately 80 nm was added to the chitosan electrolyte and homogenized in an ultrasonic bath. The Ti13Zr13Nb specimen with TiO_2_ layers served as the cathode while a platinum plate was used as the anode with an electrode spacing of 10 mm. The deposition was conducted using a DC power supply (MCP/SPN110-01C. Shanghai MCP Corp., Shanghai, China) at applied voltages of 10 V and 20 V for 1 min at room temperature. After deposition the composite coatings were rinsed with distilled water and air-dried at ambient temperature for 48 h.

#### 2.4.2. Procedure B—Chitosan–nanoHAp Coatings

The electrolytes were prepared by dispersing 1 g of high-molecular-weight chitosan (degree of deacetylation > 75%; Sigma-Aldrich, St. Louis, MO, USA) in 1 L of 1% (*v*/*v*) acetic acid (Polskie Odczynniki Chemiczne POCH, Gliwice, Poland). Separately 1 mL of Tween 20 (Polysorbate 20; Sigma-Aldrich. St. Louis, MO, USA) was diluted in 1 L of 1% acetic acid. The resulting solutions were homogenized using a magnetic stirrer at 250 rpm for 24 h at room temperature. One hour prior to electrophoretic deposition (EPD), 2.5 g/L of hydroxyapatite nanopowder (MK Nano, Mississauga, ON, Canada) with an average particle size of approximately 20 nm was added to the chitosan electrolyte and homogenized in an ultrasonic bath. The laser-treated Ti13Zr13Nb specimens served as the cathode while a platinum plate acted as the anode with an electrode spacing of 10 mm. The deposition was carried out using a DC power supply (MCP/SPN110-01C, Shanghai MCP Corp., Shanghai, China) at an applied voltage of 30 V for 1 min at room temperature. Following deposition the composite coatings were rinsed with distilled water and air-dried at ambient temperature for 48 h.

#### 2.4.3. Procedure C—nanoHAp–nanoCu Coatings

The electrophoretic deposition (EPD) was carried out using a dispersion containing 1 g of needle-shaped nanohydroxyapatite (average particle size ≈ 20 nm; MK Nano, Canada) and 0.05 g of copper nanopowder (average particle size ≈ 80 nm; Hongwu International Group Ltd., Xuzhou, China) in 1 L of 99.8% pure ethanol. The dispersion was prepared by ultrasonically mixing the nanopowders in ethanol for 1 h at room temperature. During EPD the Ti13Zr13Nb specimen with or without the TiO_2_ layer served as the cathode and a platinum plate as the anode with an electrode spacing of approximately 10 mm. The deposition was performed using a DC power supply (MCP/SPN110-01C, Shanghai MCP Corp., Shanghai, China) at an applied voltage of 30 V for 1 or 2 min at room temperature. Following deposition the specimens were air-dried for 24 h. Only the specimens with the deposited coatings (with or without the TiO_2_ layer) were subjected to heat treatment. The coated specimens were heat-treated in a high-vacuum furnace (PROTHERM PC442, Ankara, Turkey) at 800 °C for 120 min. The target temperature was reached at a controlled heating rate of 200 °C/h, after which the specimens were allowed to cool to room temperature inside the furnace chamber.

### 2.5. Characterization of Microstructure

The Ti13Zr13Nb ground alloy, nanotubular TiO_2_ layer, and coating surface were examined for each specimen with a high-resolution scanning electron microscope (SEM JEOL JSM- 7800 F, JEOL Ltd., Tokio, Japan and SEM Microscope FEI Quanta FEG 250, FEI, Hillsboro, OR, USA) equipped with an LED detector at 5 kV acceleration voltage.

### 2.6. Nanoindentation Studies

Nanoindentation tests were carried out using a NanoTest™ Vantage system (Micro Materials, Wrexham, UK) equipped with a Berkovich three-sided pyramidal diamond indenter. For specimens prepared according to Procedure A, 25 independent nanoindentation measurements were performed at a maximum load of 50 mN. For specimens obtained according to Procedure B, 5 independent measurements were conducted at a maximum load of 10 mN and for those following Procedure C, 25 independent measurements were performed at a maximum load of 5 mN. In all tests the loading and unloading times were set to 20 s with a dwell period of 5 s at maximum load. The spacing between consecutive indents was 20 μm. The load–displacement curves were analyzed using the Oliver and Pharr method [20]. Surface hardness (H) and reduced Young’s modulus (Er) were calculated using the integrated analysis software. When estimating the Young’s modulus (E) Poisson’s ratios of 0.33, 0.30, and 0.40 were assumed for the reference Ti13Zr13Nb alloy, Ti13Zr13Nb with a TiO_2_ layer, and Ti13Zr13Nb with surface modifications, respectively. All nanoindentation parameters for each specimen group are summarized in Table 3.

### 2.7. Nanoscratch Test Studies

Nanoscratch tests were performed using the same instrument and indenter as those used for the nanoindentation measurements. Each test was repeated five times under a progressively increasing load from 0 to 200 mN at a loading rate of 1.3 mN·s^−1^ over a scratch length of 500 µm. The coating adhesion was evaluated based on the critical load corresponding to an abrupt change in the frictional force recorded during the test.

## 3. Results and Discussion

**Figure 1:**
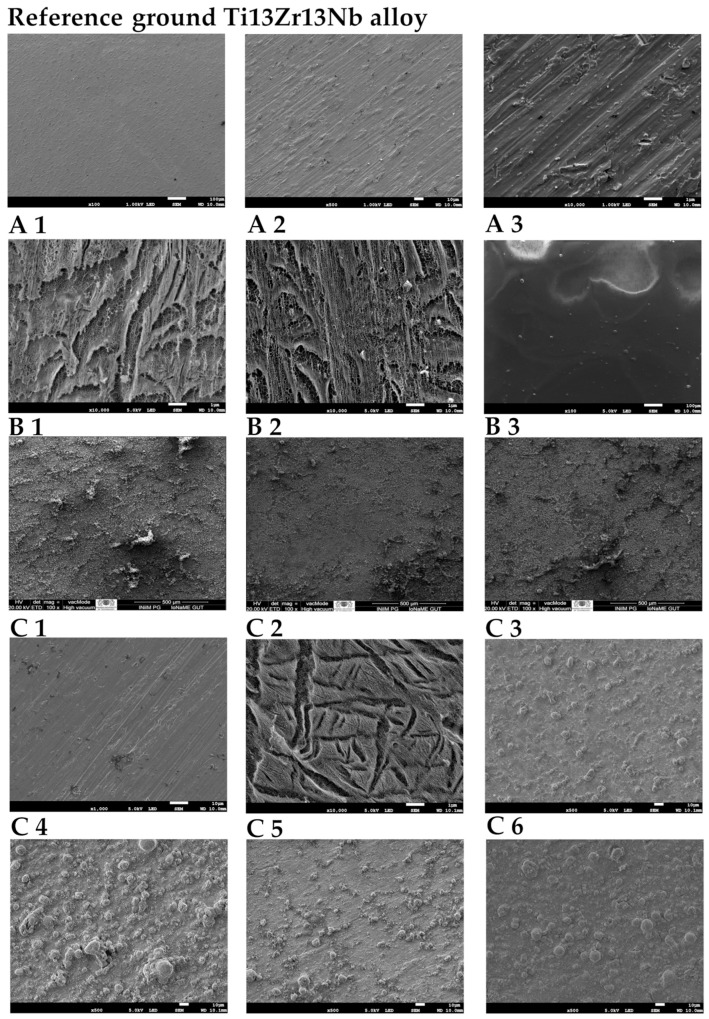
SEM micrographs of reference ground Ti13Zr13Nb alloy, chitosan–Cu (**A1**–**A3**), chitosan–HAp (**B1**–**B3**), and HAp–Cu (**C1**–**C6**) coatings deposited on Ti13Zr13Nb alloy.

Representative SEM micrographs of the coatings prepared using procedures A, B, and C are shown in Figure 1. Obvious differences in surface morphology can be observed, depending on suspension and deposition parameters. For chitosan–Cu coatings (series A), at lower voltages of deposition, the TiO_2_ nanotubular substrate is still visible. For higher voltages (A3), the nanotubular structure disappeared due to a greater thickness of the coating (throughout this study, all references to ‘thicker’ or ‘thinner’ coatings are comparative and qualitative—they reflect expected trends from applied voltage and deposition time, and also from observed surface morphology). Chitosan–HAp coatings (series B) are characterized by a more heterogeneous structure with visible granular agglomerates of hydroxyapatite embedded in the polymer matrix. Their surface is much rougher compared with series A. No evident influence of laser treatment on the homogeneity or microstructure of the analyzed surfaces was observed. In turn, the HAp–Cu coatings (series C) have a denser and more uniform microstructure with visible agglomerates of nanohydroxyapatite. Only for coatings from group C5 were characteristic polishing lines visible, testifying to a low thickness of modification. An increase in deposition time from 1 to 2 min results in a higher concentration of nano-HAp agglomerates on the surface of the coatings. For sample C6, small surface cracks can be observed, which probably formed due to thermal shrinkage during heat treatment.

The results of the nanoindentation test—the maximum depth of indentation, hardness, Young’s modulus, H/E ratio, and plastic-to-elastic work ratio for all tested samples—are presented in Table 4.

**Figure 2:**
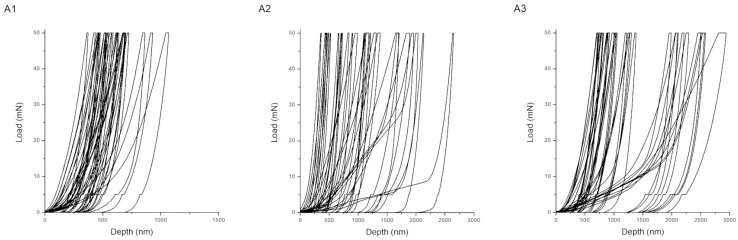
Load–displacement curves for samples obtained according to procedure A.

In this respect, it has to be pointed out that the relatively high standard deviations of some nanoindentation data sets (in particular, A2, A3, B2–B3, and C3–C6, Table 4) do not originate from experimental artifacts but instead reflect the intrinsic microstructure of the coatings. As already illustrated by the SEM images (Figure 2) and the 3D hardness/modulus maps (Figures 5 and 6), porosity, agglomerates of either HAp or Cu nanoparticles, and thickness variations locally occur in the coatings, thus leading to spatial variations in the mechanical response. Due to the fact that the indents were placed at randomly selected positions, scatter in nanoindentation data reflects the real heterogeneity of the coating systems. Even despite this variability, the differences between the coating series A, B, and C remain clearly distinguishable.

For coatings from series A, deposited from chitosan–Cu suspensions, the nanomechanical behavior strongly depended on the applied deposition voltage and the presence of a surfactant. The A1 coating (anodized Ti13Zr13Nb substrate, EPD at 10 V for 1 min) exhibited the highest hardness (8.21 ± 3.83 GPa) and Young’s modulus (157.49 ± 61.31 GPa), corresponding to an H/E ratio of 0.053. The relatively low Wp/We value (1.15) indicates a favorable balance between elasticity and plasticity, typical of compact, well-adhered coatings. When Tween 20 was introduced into the suspension (A2), the average hardness and modulus decreased to 4.73 ± 5.31 GPa and 85.15 ± 71.77 GPa, respectively, while Wp/We increased to 1.34. The larger standard deviation in both H and E values suggests local softening and a less uniform structure, likely due to the influence of the surfactant on particle aggregation during deposition. For the coating deposited at a higher voltage (A3, 20 V, 1 min), both hardness (2.43 ± 1.90 GPa) and Young’s modulus (38.44 ± 20.90 GPa) decreased, accompanied by similar Wp/We values (1.34). This indicates a transition from a compact coating to a more porous structure, consistent with gas evolution effects at higher voltages. Overall, the A series coatings exhibited a trade-off between mechanical strength and homogeneity. Low-voltage deposition (A1) provided the best mechanical performance, while higher voltages or surfactant addition increased duc-tility but reduced coating integrity.

Coatings from series B, with hydroxyapatite nanoparticles, gave remarkably lower hardness values ranging from 0.05 to 0.13 GPa and modulus values between 3.16 and 5.09 GPa, which were uninfluenced by surface treatment. Calculated H/E ratios between 0.01 and 0.04 and Wp/We values between 4.06 and 19.04 evidence the predominance of plastic deformation during indentation. Laser-treated samples B2 and B3 exhibited the highest indentation depths for the Wp/We ratio > 15, indicating more ductile behavior and thus weak mechanical bonding between the coating and the substrate. The general lower hardness of the B series coatings can be explained by the polymeric nature of the chitosan matrix and the presence of hydroxyapatite particles. While hydroxyapatite is bioactive, it may also act as a stress concentrator and diminish the cohesive strength of the composite film.

Coatings containing both Cu and HAp nanoparticles (series C) showed intermediate mechanical properties between the A and B series. Hardness and Young’s modulus values for these coatings ranged, depending on the surface treatment and deposition voltage, between 0.10 and 2.91 GPa and between 12.41 and 89.53 GPa, respectively. H/E ratios within 0.02–0.03 and Wp/We within 3.5–4.7 suggest improved elastic recovery in comparison with pure HAp-containing coatings and lower stiffness in comparison with Cu-only coatings. Coatings deposited onto anodized substrates (C1 and C2) demonstrated the most favorable balance between hardness and ductility. It appears that this surface treatment promotes interfacial bonding due to the specific nanostructure of the TiO_2_ layer formed by this process. Coatings deposited directly onto unmodified titanium (C3–C6) were softer and more heterogeneous (H/E < 0.01 and Wp/We > 10), corresponding to a more compliant but mechanically weaker structure.

As the results demonstrate, chitosan–Cu coatings presented the highest hardness and Young’s modulus with a compact and elastic layer well bonded to the anodized TiO_2_ surface. In contrast, chitosan–HAp coatings were softer and more ductile, exhibiting a predominantly plastic deformation behavior and, therefore, a limited load-bearing capacity. Finally, the nanoHAp–nanoCu coatings displayed the highest critical delamination up to one order of magnitude higher than for single-component systems, indicative of superior cohesion and interfacial toughness. Such results are significant evidence that the application of nanoparticles is highly important for mechanical behavior demonstrated by hardness and strength, toughness, elasticity, and also adhesion to the base. These results are relatively novel; some earlier investigations support these observations, and some others do not.

In [21], the compressive strength of HAp was 1570 MPa and that of HAp-Chi-PVA was 1893 MPa. The bonds between polymer and ceramic were suggested to be weak; therefore, the application of nanomaterials should result in a much better mechanical strength, as observed here. However, in [22] the scaffolds containing 33% HAp expressed values almost two times higher in comparison with the scaffolds with 50% HAp, suggesting that the application of nanomaterials should again be positive. In [23] in tensile tests carried out for nylon/chitosan/HAP biocomposites, a considerable decrease in maximum load was observed compared with the base material, which was in accordance with previous statements on the bond between ceramic and polymer components. Moreover, the addition of TiO_2_ increased the mechanical strength and Young’s modulus. On the other hand, in [24], the addition of quaternary chitosan to HAp increased the adhesion strength between the coatings and the substrate. In [25] for a chitosan–HAp hydrogel the prominent effect of process parameters on the mechanical strength was noticed. In [26] it was demonstrated that the addition of nanoparticles had a significant impact on the tensile strength and percentage for a chitosan–HAp composite. In [27] the enhanced interfacial interactions between the polymer matrix and the nanofiller in the composite film contributed to its robustness and durability. Namely, the ceramic nanofiller enhances stiffness and load bearing.

**Figure 3:**
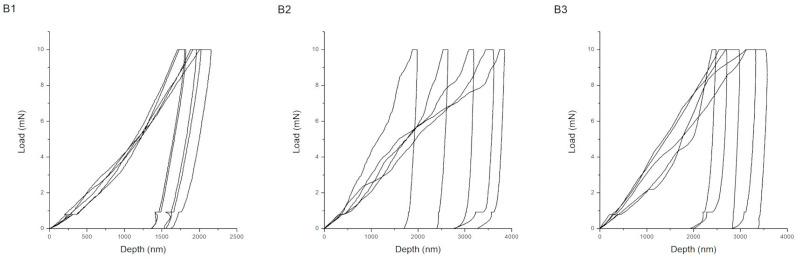
Load–displacement curves for samples obtained according to procedure B.

**Figure 4:**
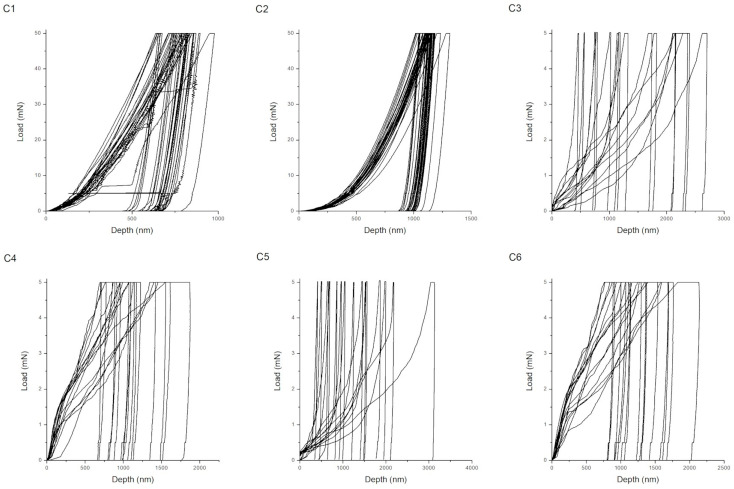
Load–displacement curves for samples obtained according to procedure C.

Representative load–displacement (P–h) curves obtained during nanoindentation for coatings produced according to procedures A, B, and C are shown in Figure 2, Figure 3 and Figure 4. The shape and depth of the indentation curves reflect the coating stiffness, plasticity, and adhesion, providing direct insight into their nanomechanical behavior. For the chitosan–Cu coatings (series A), the curves show steep loading slopes and relatively short unloading branches, characteristic of hard and moderately elastic materials. Sample A1 (10 V, anodized substrate) exhibits the smallest maximum indentation depth, which corresponds to its high hardness of 8.21 ± 3.83 GPa and compact structure. The presence of a distinct unloading segment indicates good elastic recovery and strong interfacial bonding to the anodized TiO_2_ layer. Upon the addition of surfactant (Tween 20), the slope of the loading curve decreases for A2 and the maximum indentation depth increases, indicating partial softening of the coating and less homogeneous compaction. At a higher deposition voltage (A3, 20 V), the curve becomes more rounded with deeper penetration and less pronounced unloading, which corresponds to increased plastic deformation and possible microporosity caused by gas evolution during EPD. Therefore, the mechanical response varies from stiff–elastic (A1) to a more ductile and compliant one (A3). In the case of hydroxyapatite coatings with nanoCu (series C) that contained both Cu and HAp nanoparticles, the load–displacement curves combined features of the two previous systems. Specimens C1 and C2 exhibited moderately steep loading curves and distinct elastic unloading behavior. These corresponded to intermediate hardness values (0.10–2.91 GPa) and a balanced ratio of elastic to plastic deformation (Wp/We ≈ 3.5–4.7). These samples showed improved elastic recovery and a higher cohesive strength that, in the case of sample C2, can be assigned to the nanostructured TiO_2_ layer formed upon anodization. To better explain these relations, it has to be underlined that specific features of the load–displacement curves correspond to the particular mechanical parameters of the coatings. Coating stiffness is mirrored primarily by the slope of the loading segment and by the pronounced elastic response during unloading; indeed, steeper loading slopes and shorter elastic recovery segments, as observed for the chitosan–Cu coatings (A series), are in good agreement with the higher hardness and Young’s modulus of the mentioned coatings. Plasticity is mirrored by the maximum indentation depth and by the extent of a plastic tail in the unloading portion of the curve, as is apparent for chitosan–HAp coatings (B series) exhibiting wide and shallow curves with a gradual unloading shape, in good correspondence with their high Wp/We ratio and low H/E value. Coatings deposited on anodized substrates (C5) show more defined unloading slopes, in agreement with their higher critical delamination loads. For series C, the high Lc values also result from the synergistic action of the hybrid HAp–Cu composition and heat treatment. Figure 2, Figure 3 and Figure 4 can be more directly compared, further reinforcing the correspondence between the curve morphology and the mechanical behavior.

In contrast, the C3–C6 coatings exhibit deeper indentation depths, longer plastic tails on their unloading curves, and consequently have a lower mechanical stability and weaker interfacial bonding. This behavior is consistent with their low hardness and H/E ratio values (<0.02).

In more detail, from a biomedical perspective, higher H/E ratios reflect better elastic strain tolerance and resistance to cracking and wear, while a lower Wp/We characterizes reduced susceptibility to plastic deformation and an increased cyclic durability of coated implants [18,28,29]. Coatings for load-bearing applications must balance stiffness, elasticity, and interfacial strength to minimize micromotion and ensure long-term osseointegration [30,31]. In this context, the chitosan–Cu coatings, especially A1, and nanoHAp–nanoCu coatings deposited on anodized substrates (C1–C2) show more favorable H/E and Wp/We ratios and significantly higher critical delamination loads than the chitosan–HAp coatings of series B. From a mechanical design point of view, coatings for load-bearing implant applications should be characterized by intermediate H/E ratios ideally on the order of 0.02–0.05 for polymer/ceramic bioactive layers and relatively low to moderate Wp/We values, approximately 1–5, that have an optimal balance between elastic strain tolerance and permanent damage resistance [15,32]. In this respect, the chitosan–Cu (series A) and ground Ti13Zr13Nb and anodized substrates (C1–C2) present more favorable H/E and Wp/We combinations than the highly plastic chitosan–HAp coatings, series B, whose very high Wp/We ratios (>10) and low H/E values indicate poor load-bearing capability. These properties point to improved resistance against micromotion and interfacial failure. The presence of Cu nanoparticles (coatings C series) provides, in addition to the antibacterial and potential osteogenic advantages, improved cohesive strength and interfacial toughness upon incorporation in a chitosan or HAp matrix and support by an anodized TiO_2_ interlayer.

In contrast, the indentation behavior of the chitosan–HAp coatings (series B) is distinctly different; their wide, shallow curves display gradual unloading paths typical of soft, highly plastic materials. The maximum indentation depths are considerably greater compared with those for series A, reflecting low hardness values in the 0.05–0.13 GPa range and a high plastic contribution to the total deformation (Wp/We up to 19). Even broader curves are observed for the laser-treated coatings (B2, B3), indicating more extensive plastic flow and hence lower resistance to localized deformation, in accordance with the SEM observations of porous and melted surface regions. This behavior confirms that laser treatment enhances the ductility of the surface but deteriorates the coating’s load-bearing capacity and stiffness.

**Figure 5:**
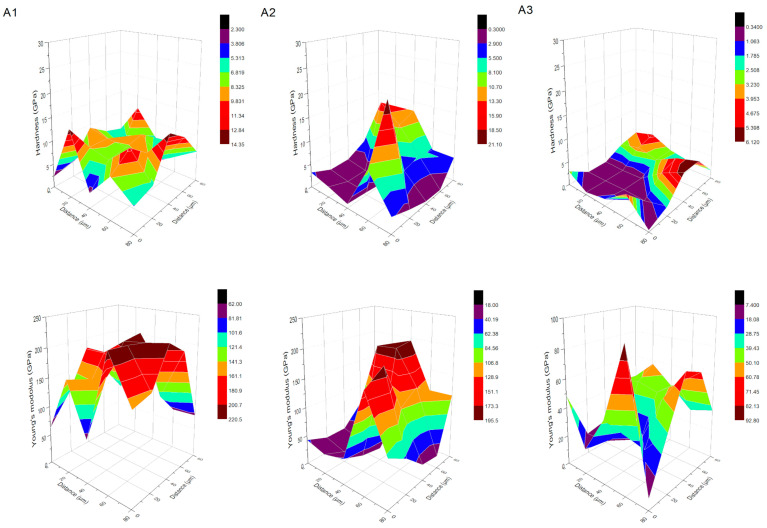
Three-dimensional distributions of hardness and Young’s modulus for samples obtained according to procedure A.

**Figure 6:**
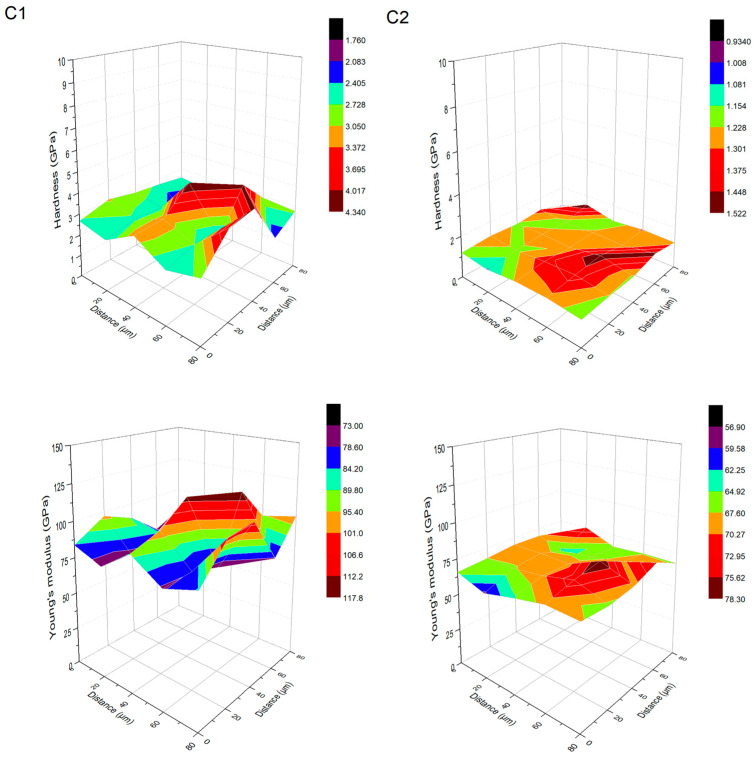
Three-dimensional distributions of hardness and Young’s modulus for ground Ti13Zr13Nb and anodized Ti13Zr13Nb.

The 3D maps of hardness and Young’s modulus for the coatings analyzed in this work highlight distinct variations in the surface uniformity and local mechanical response as a function of deposition parameters and surface treatment (Figure 5 and Figure 6). For chitosan–Cu coatings (series A, A1–A3), a moderate homogeneity of the mechanical properties is observed for the coating deposited at 10 V on anodized Ti13Zr13Nb (A1), whereas the addition of surfactant (A2) and/or increased deposition voltage up to 20 V (A3) induce progressive heterogeneity. Sample A1 shows relatively flat plateaus with similar H and E values and with small local fluctuations (‘hard islands’) which correspond to the highest average hardness and modulus and to the smallest plastic work ratio (Wp/We ≈ 1.15). On the contrary, A2 and A3 present extended soft regions along with stronger H and E gradients, in agreement with reduced hardness and stiffness and higher variability (high SD), characteristic of thicker, less compact coatings showing local porosity or Cu nanoparticle agglomeration under strong EPD conditions. The scanning electron microscopy images of sample A3 show a homogeneous and compact chitosan structure, but with well distinguishable agglomerates of copper nanoparticles. Such microstructural uniformity would lead to a more homogeneous hardness distribution, with the Young’s modulus showing only a few isolated, very local increases, whereas its variation, at the micrometer scale, is limited. Sample A1 and sample A2 presented a different behavior, with the scanning electron microscopy images indicating the presence of a more developed microstructure characterized by some exposed regions of nanotubular substrate and several agglomerates of Cu nanoparticles. The presence of those morphological features increases the variability in the local mechanical response, as shown by the greater scattering of the hardness and Young’s modulus distributions

Specimen Ti13Zr13Nb and anodized Ti13Zr13N (Figure 6) show more uniform plateaus of H and E with smaller local contrasts, reflecting balanced H/E ratios (≈0.02–0.03) and moderate Wp/We values (≈3.5–4.7), which result from effective mechanical anchoring in the nanostructured TiO_2_ layer. For both the Ti13Zr13Nb and anodized Ti13Zr13Nb substrates, a strong correlation is obtained between the 3D mechanical maps and SEM morphology. In the case of the anodized substrate, SEM analysis reveals a regular nanotubular TiO_2_ layer. This geometrical configuration enlarges the specific surface area and offers effective mechanical anchorage. Accordingly, hardness and modulus maps present uniform H and E plateaus, with low local contrasts. The resulting H/E ratios as well as Wp/We values indicate the stable, elastoplastic behavior of the layer. Mechanical distributions are highly uniform due to the complete lack of discontinuities or structural defects in the SEM images. Finally, for the non-anodized Ti13Zr13Nb substrate, the smooth and compact topography, as depicted by SEM, corresponds to uniform H and E maps, which further strengthens the microstructure–mechanical response relationship.

The nanoscratch tests evidenced well-defined, process-dependent differences in coating adhesion, quantified by the critical normal load Lc at delamination. In the case of chitosan–Cu coatings, from series A, Lc consistently increased with deposition voltage/surfactant use (A1: 24.32 ± 8.08 mN; A2: 31.82 ± 8.36 31.82; A3: 53.67 ± 19.52), which was accompanied by enhanced friction loads to failure, from ~39 up to ~87 mN, consistent with a transition from compact but thinner films, A1, to thicker, more compliant layers, A3, which allow for higher tangential stresses before catastrophic spallation. Chitosan–HAp coatings, from series B, showed similar or even lower Lc (B1–B3: ~22–30 mN) but higher friction forces to failure, ~69–83 mN, indicating pronounced plastic dissipation before delamination, consistent with their high Wp/We ratios and low hardness. On the contrary, nanoHAp–nanoCu coatings deposited without or with previous anodization at 30 V, series C, C3–C6, displayed an order-of-magnitude higher adhesion, with Lc in the range of 150–173 mN, while friction forces to failure remained moderate, ~63–77 mN. The markedly elevated Lc for C3–C6 points to a beneficial synergistic effect of the hybrid phase and of the EPD/thermal schedule on the interfacial toughness and cohesive strength of the film, whereas the A and B systems fail at substantially lower normal loads, consistent with their lower hardness/modulus and higher plasticity observed using nanoindentation. In [33] the excellent adhesion of chitosan–HAp coatings was observed. In [34] a similar behavior was observed, but with a slight worsening of adhesion with the addition of HAp. In [35] the tensile strength of pure chitosan was 13.50 MPa, while for Ag–HAp–chitosan, evidently higher strength values were found. As in many protective coatings, the interpretation of Lc in terms of the dominant failure mode (cohesive cracking within the coating vs. adhesive delamination at the coating–substrate interface) follows established methodologies. In these scenarios, friction force evolution is correlated to the initiation of spallation or interfacial failure. Similar methods have been used for high-entropy Zr–Si-based coatings, as found by Guo et al. [36]. The more gradual friction force rise and higher Lc values from nanoHAp–nanoCu coatings indicate enhanced cohesive strength and improved interfacial toughness.

**Figure 7:**
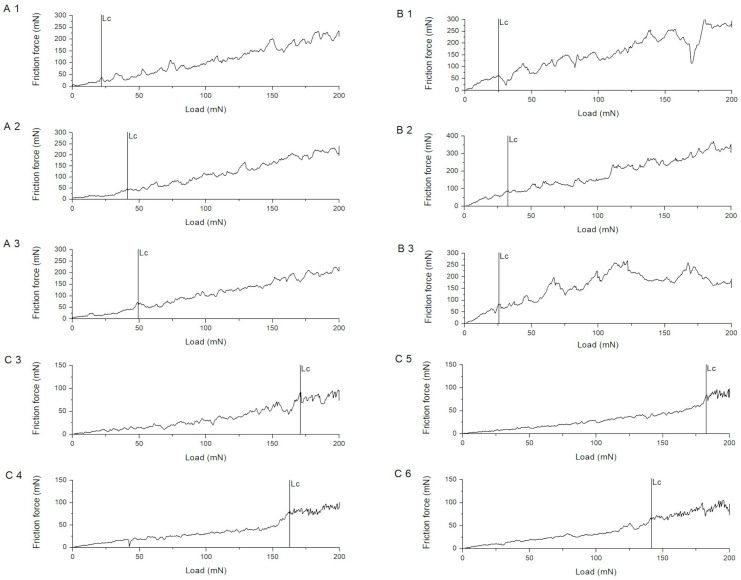
Normal load–friction force curves obtained from single scratch tests for samples of different coating groups, showing the critical delamination load (Lc).

The results of the nanoscratch tests are tabulated in Table 5. The graphs for individual samples from the respective groups are plotted as friction force against normal force and are presented in Figure 7.

## 4. Conclusions

The current research indicates that the nanomechanical and adhesive properties of chitosan-based composite coatings deposited by EPD on the surface of a Ti13Zr13Nb alloy are significantly controlled by the interactions between surface pre-treatments, suspension composition, and EPD processing parameters. Anodization was an excellent method for surface modification, leading to homogeneous coating development, increased elastic recovery, and better interfacial bonding due to the presence of nanotubular TiO_2_. In contrast, laser surface modification enhanced the ductility of the deposited coatings at the cost of reduced stiffness and homogeneity, especially in the case of chitosan–HAp systems.

Chitosan–Cu coatings (series A) presented the highest hardness (up to 8.2 GPa) and Young’s modulus (≈157 GPa), with a compact and elastic layer well bonded to the anodized TiO_2_ surface. In contrast, chitosan–HAp coatings (series B) were softer and more ductile, exhibiting a predominantly plastic deformation behavior (Wp/We > 10) and, therefore, a limited load-bearing capacity. Nanoscratch tests showed that nanoHAp–nanoCu coatings displayed the highest critical delamination loads (150–173 mN), up to one order of magnitude higher than for single-component systems, indicative of superior cohesion and interfacial toughness. In general, the present results illustrate that a combination of anodization with optimized electrophoretic deposition parameters—voltage and time—along with the incorporation of nanoparticles (nanoHAp + nanoCu) is an effective strategy to control the nanomechanical and adhesive properties of titanium-based biofunctional coatings.

The direct relevance of these insights applies to the design of advanced surface-engineered biomaterials intended for long-term implantation, where the balance between stiffness, plasticity, and adhesion is crucial for resisting micromotion, wear, and delamination in vivo.

## Figures and Tables

**Table 1:** The chemical composition of the Ti13Zr13Nb alloy. wt.%.

Element	Nb	Zr	Fe	C	N	O	Ti
wt.%	13.0	13.0	0.05	0.04	0.019	0.11	remainder

**Table 2:** Details of surface modifications applied in various procedures.

Procedure	Substrate	Electrochemical Oxidation	Laser Treatment	Coating Deposition Parameters
A1	Ti13Zr13Nb	H_3_PO_4_ + HF; 20 V; 20 min	none	1 g/L chitosan + 0.05 g/L CuNPs; 10 V; 1 min
A2				1 g/L chitosan + 0.05 g/L CuNPs + Tween20; 10 V; 1 min
A3				1 g/L chitosan + 0.05 g/L CuNPs + Tween20; 20 V; 1 min
B1	Ti13Zr13Nb	none	none	1 g/L chitosan + 2.5 g/L HApNPs + Tween20; 30 V; 1 min
B2			750 W; pulse duration 3.25 ms; frequency 25 Hz	
B3			1500 W; pulse duration 3.25 ms; frequency 25 Hz	
C1	Ti13Zr13Nb	none	none	none
C2		H_3_PO_4_ + HF; 20 V; 20 min		none
C3		none		1 g/L HApNPs + 0.05 g/L CuNPs + Tween20; 10 V; 1 min
C4				1 g/L HApNPs + 0.05 g/L CuNPs + Tween20; 20 V; 1 min
C5		H_3_PO_4_ + HF; 20 V; 20 min		1 g/L HApNPs + 0.05 g/L CuNPs + Tween20; 10 V; 1 min
C6				1 g/L HApNPs + 0.05 g/L CuNPs + Tween20; 20 V; 1 min

**Table 3:** Details of nanoindentation tests parameters.

Procedure	Number of Indentations	Distance Between Indentation [µm]	Maximum Force [mN]	Loading; Dwell with Maximum Force and Unloading [s]	Poisson’s Ratio for Analyzes [-]
A1–A3	25	20	50	20 s–5 s–15 s	0.261
B1–B3	5		10		0.3
C1–C2	25		50		Ti13Zr13Nb—0.3; TiO_2_—0.25
C3–C6	15		5		0.25

**Table 4:** Nanoindentation results.

Procedure	Maximum Depth of Indentations (nm)	Hardness (GPa)	Young’s Modulus (GPa)	H/E Ratio (-)	Wp/We Ratio (-)
A1	619 ± 161	8.21 ± 3.83	157.49 ± 61.31	0.053 ± 0.017	1.15 ± 0.26
A2	1170 ± 622	4.73 ± 5.31	85.15 ± 71.77	0.048 ± 0.018	1.34 ± 0.75
A3	1487 ± 714	2.43 ± 1.90	38.44 ± 20.90	0.055 ± 0.023	1.34 ± 0.61
B1	1954 ± 147	0.13 ± 0.02	3.16 ± 0.29	0.042 ± 0.005	4.06 ± 0.61
B2	3058 ± 756	0.06 ± 0.03	5.09 ± 1.09	0.010 ± 0.004	15.71 ± 4.41
B3	3017 ± 44	0.05 ± 0.02	4.69 ± 0.77	0.010 ± 0.002	19.04 ± 10.06
C1	796 ± 78	2.91 ± 0.67	89.53 ± 13.27	0.032 ± 0.004	3.54 ± 0.55
C2	1144 ± 57	1.26 ± 0.13	68.27 ± 4.70	0.018 ± 0.001	4.75 ± 0.23
C3	1499 ± 574	0.10 ± 0.08	25.81 ±16.38	0.004 ± 0.001	25.17 ± 13.05
C4	1168 ± 325	0.14 ± 0.07	18.41 ± 11.16	0.008 ± 0.003	24.64 ± 10.36
C5	1397 ± 395	0.10 ± 0.06	23.95 ± 8.24	0.003 ± 0.002	12.01 ± 4.70
C6	1343 ± 360	0.10 ± 0.05	12.41 ± 4.77	0.008 ± 0.004	24.10 ± 8.62

**Table 5:** Nanoscratch test results.

Procedure	Critical Normal Force (mN)	Critical Friction Force (mN)
A1	24.32 ± 8.08	39.23 ± 10.43
A2	31.82 ± 8.36	40.22 ± 9.64
A3	53.67 ± 19.52	86.85 ± 20.39
B1	27.22 ± 9.67	70.89 ± 24.69
B2	29.64 ± 4.80	83.35 ± 12.90
B3	21.68 ± 4.83	68.99 ± 14.98
C3	165.04 ± 7.87	63.10 ± 5.17
C4	158.31 ± 11.64	77.04 ± 7.35
C5	172.95 ± 25.97	67.33 ± 30.29
C6	149.73 ± 39.32	70.89 ± 18.76

## Data Availability

The original contributions presented in this study are included in the article. Further inquiries can be directed to the corresponding author.
